# Clinicopathological characteristics and prognostic outcomes in consecutive Japanese patients with high microsatellite-instability colorectal cancer: real-world data from universal screening

**DOI:** 10.1186/s12876-026-04696-7

**Published:** 2026-03-13

**Authors:** Akira Inoue, Yujiro Nishizawa, Masahiro Hashimoto, Yuki Ozato, Yoshihiro Morimoto, Kenta Furukawa, Masashi Hirota, Yasuhiro Miyazaki, Akira Tomokuni, Masaaki Motoori, Kazumasa Fujitani

**Affiliations:** Department of Gastroenterological Surgery, Osaka General Medical Center, 3-1-56 Mandaihigashi, Sumiyoshi-ku, Osaka City, Osaka 558-8558 Japan

**Keywords:** Asian populations, Colorectal cancer, High microsatellite instability, Real-world data, Universal screening

## Abstract

**Background:**

High microsatellite instability (MSI-H) is an established prognostic marker in various cancers, including colorectal, gastric, and endometrial cancers. However, comprehensive real-world data from universal screening in Asian populations remain limited. We aimed to evaluate the clinicopathological and prognostic impact of MSI-H status in Japanese patients with colorectal cancer (CRC).

**Methods:**

This retrospective cohort study was conducted at Osaka General Medical Center, Japan. A total of 346 consecutive patients with CRC who underwent MSI testing between January 2021 and December 2022 were retrospectively analyzed. MSI status and associated clinicopathological features were assessed. Overall survival (OS) and recurrence-free survival (RFS) were analyzed using Kaplan–Meier survival curves and Cox regression models.

**Results:**

Among the 346 patients, 29 (8.4%) had MSI-H tumors, which were more frequently right-sided (*p* <0.0001) and poorly differentiated (*p* <0.0001) compared with non-MSI-H. Genomic analysis revealed that MSI-H tumors had significantly fewer *RAS* mutations (*p* = 0.024) and more *BRAF* mutations (*p* <0.0001) compared with non-MSI-H tumors. Survival analysis showed no significant differences in RFS (HR, 0.67; 95% CI, 0.26–1.90; *p* = 0.40) or OS (HR, 0.64; 95% CI, 0.30–1.58; *p* = 0.78) between patients with MSI-H and non–MSI-H tumors. Multivariate analysis identified MSI-H as a potential independent predictor of favorable OS (HR, 0.07; 95% CI, 0.005–0.90; *p* = 0.04), whereas *BRAF* mutations were associated with poor OS (HR, 7.39; 95% CI, 1.32–41.50; *p* = 0.02).

**Conclusions:**

This real-world study suggests that MSI-H status is associated with distinct clinicopathological characteristics and may be associated with improved overall survival in Japanese patients with CRC undergoing universal MSI screening. These findings support the clinical relevance of MSI testing in routine clinical practice and highlight the need for further validation in a larger cohort.

## Background

Colorectal cancer (CRC) remains a significant cause of cancer-related deaths worldwide, necessitating ongoing efforts to refine diagnostic and treatment approaches [1]. Advances in molecular profiling have revealed substantial heterogeneity within CRC, with distinct genetic subgroups influencing prognosis and therapeutic response. One of these subgroups, characterized by high microsatellite instability (MSI-H) resulting from deficient mismatch repair, has garnered considerable attention for its unique clinicopathological features and its role as a predictive biomarker for immune response and treatment efficacy [2–4]. MSI-H CRC, distinguished by a high mutation load and pronounced immunogenicity, is consistently associated with a favorable prognosis, especially in early-stage disease, and demonstrates a strong response to immune checkpoint inhibitors [5–8]. These findings underscore the critical role of MSI-H in guiding personalized treatment strategies for CRC.

MSI-H testing also plays a critical role in identifying Lynch syndrome (LS), a hereditary condition that significantly increases the risk of CRC and other malignancies. Universal MSI screening for all newly diagnosed patients with CRC is increasingly recommended to improve LS detection rates, enabling targeted surveillance and preventive interventions for affected individuals and their families [9, 10]. However, despite the established significance of MSI status, most supporting evidence originates from Western populations and clinical trial data, often excluding real-world clinical settings and diverse demographic backgrounds [4, 6, 7]. Consequently, although MSI-H is recognized as a favorable prognostic factor in CRC, its impact remains underexplored in Asian populations, where genetic, environmental, and dietary factors may influence CRC biology. Additionally, many studies on MSI-H have focused on selective patient cohorts, potentially limiting their applicability to routine clinical practice. These gaps highlight the need to evaluate the prognostic relevance of MSI status in Asian populations under real-world clinical conditions, bridging the divide between research findings and practical applications.

To address these gaps, we aimed to evaluate the clinicopathological and prognostic impact of MSI-H status in a cohort of consecutive Japanese patients with CRC who underwent universal MSI testing in a real-world setting. By analyzing a comprehensive dataset from routine clinical care, this study provides valuable insights into the role of MSI status as an independent prognostic factor for CRC survival outcomes in an unselected and consecutively treated Japanese population. Importantly, our approach differs from prior research by incorporating real-world data that reflect everyday clinical practice and patient diversity. This enhances the generalizability of our findings and contributes a novel dataset for the global understanding of the clinical implications of MSI-H.

## Methods

### Study design and patient selection

This retrospective study included 346 consecutive patients with CRC who underwent MSI testing at Osaka General Medical Center between January 2021 and December 2022. The patient inclusion criteria were confirmed pathological diagnosis of CRC and available MSI testing data. Clinical staging was determined using endoscopy, multi-slice computed tomography (CT), and magnetic resonance imaging (MRI). Lymph nodes with a short-axis diameter of ≥ 10 mm on CT or MRI or high-intensity signals on positron emission tomography images were considered indicative of metastasis. Staging followed the Japanese Classification of Colorectal, Appendiceal, and Anal Carcinoma: the 3rd English Edition [11].

### Universal screening for LS via MSI testing

All patients with CRC underwent universal MSI testing to screen for LS, regardless of clinical stage or treatment approach. MSI testing was conducted using a polymerase chain reaction-based assay with a standard panel of mononucleotide microsatellite markers (BAT-25, BAT-26, NR-21, NR-24, and MONO-27), following established protocols​ [12]. All tests were performed using the same platform at a single institutional laboratory to ensure methodological consistency. Tumors were classified as MSI-high (MSI-H) when instability was detected in two or more markers. Tumors showing instability in only one marker were classified as MSI-low and grouped with non-MSI-H tumors for analysis, consistent with previous clinical studies [7, 8]. Cases with technically inconclusive results were re-evaluated, and no cases with unresolved MSI status were included in the final analysis.

Genetic testing for germline mismatch repair (*MMR*) gene mutations—essential for a definitive diagnosis of LS—was not routinely performed in this study owing to the lack of national health insurance coverage in Japan.

### Follow-up and survival analyses

Patients were monitored every 3 months for the first 3 years postoperatively and every 6 months thereafter, up to 5 years. Tumor markers carcinoembryonic antigen and carbohydrate antigen 19 − 9 were measured at each follow-up visit. CT scans were performed every 6 months, and a total colonoscopy was conducted annually.

Recurrence-free survival (RFS) was analyzed in 268 patients with CRC without distant metastasis who underwent curative resection, based on recurrence events observed during follow-up. Overall survival (OS) analysis included all 346 patients, with survival status verified through medical records and follow-up visits.

### Ethics statement

This study was approved by the Institutional Review Board of Osaka General Medical Center (approval number: 2020-069). Informed consent was obtained using an opt-out approach, allowing patients to decline participation by contacting the institute through information provided on our website. This study was conducted in accordance with the principles of the Declaration of Helsinki.

### Statistical analyses

Continuous variables are reported as mean and standard deviation, or as median and interquartile range. Between-group comparisons (MSI-H vs. non-MSI-H) were conducted using the Student’s *t*-test, Wilcoxon rank-sum test, or Pearson chi-squared test, as appropriate. Survival estimates were calculated using the Kaplan–Meier method, with statistical significance assessed via the log-rank test. Cox proportional hazards regression models were used to calculate hazard ratios (HRs) and 95% confidence intervals (CIs) for survival outcomes. Variables included in the multivariate Cox regression models were selected a priori based on established clinical relevance criteria and prior literature, rather than univariate screening. To minimize the risk of overfitting, only a limited number of clinically important variables were included in each model. The variables considered included age, sex, tumor size, pathological stage, MSI status, and *BRAF* mutation status—depending on the outcome of interest. Given the limited number of survival events, particularly among MSI-H patients, the number of covariates in the multivariable models was intentionally restricted.

All statistical analyses were performed using R software (R Foundation for Statistical Computing, Vienna, Austria) and GraphPad Prism (version 6.01 for Windows; GraphPad Software, San Diego, CA, USA). A two-tailed *p*-value < 0.05 was considered statistically significant.

## Results

### Clinicopathological characteristics and MSI status

A total of 346 patients with CRC who underwent MSI testing at our medical center between January 2021 and December 2022 were analyzed. The association between clinicopathological characteristics and MSI status is summarized in Table 1. Among these patients, 29 (8.4%) were identified as having MSI-H tumors. Patients with MSI-H tumors demonstrated significantly different characteristics compared with those with non-MSI-H tumors. Specifically, MSI-H tumors were more frequently located on the right side (*p* < 0.0001) and exhibited poorly differentiated histology (*p* < 0.0001). Although not statistically significant, patients with MSI-H tumors showed a higher prevalence of family history of CRC and/or other malignancies. No significant differences were observed between the groups regarding median age, sex, tumor size, or TNM stages. Regarding genomic mutational status, MSI-H tumors were associated with fewer *RAS* mutations (*p* = 0.024) and more *BRAF* mutations (*p* < 0.0001) compared with non-MSI-H tumors. Notably, *BRAF* mutations were observed in 10 of 29 patients with MSI-H tumors (34.5%), whereas only 6 of 317 patients with non-MSI-H tumors (1.9%) harbored *BRAF* mutations, indicating a pronounced imbalance between the two groups. Among the 346 patients with CRC, 312 (90.2%) underwent surgical resection, whereas 34 (9.8%) received systemic chemotherapy alone because of unresectable or metastatic disease. Among the 29 patients with MSI-H tumors, 4 (13.8%) received immune checkpoint inhibitors as part of their treatment. No cases with unresolved or equivocal MSI results remained after repeat testing, and all patients included in the analysis had definitive MSI classification.

**Table 1:** Clinico pathological findings in relation to the MSI status

Patient characteristics	MSI-H CRC(n = 29)	Non-MSI-H CRC(n = 317)	*p*-value
Age (years) (range)	76 (37–93)	74 (34–93)	0.56
Sex			
Men	16	178	0.92
Women	13	139	
Patient history of CRC and/or other cancers			
Present	9	94	0.84
Absent	20	223	
Family history of CRC and/or other cancers			
Present	14	101	0.098
Absent	15	216	
Tumor location			
Right	22	86	<0.0001
Left	7	231	
Tumor size, mm (range)	45 (12–115)	45 (5–150)	0.71
Tumor depth			
Tis, T1, T2	10	61	0.089
T3, T4	19	256	
Lymph node metastases			
N0	19	163	0.18
N1-2	10	154	
Distant metastases			
Present	2	69	0.058
Absent	27	248	
UICC stage			
0	0	3	0.063
I	9	47	
II	10	98	
III	8	100	
IV	2	69	
Tumor histology			
tub1/tub2	19	302	<0.0001
por/muc/sig/others	10	15	
Genomic status			
* RAS *(wild/mutant/unknown)	21/6/2	154/149/14	0.024
* BRAF *(wild/mutant/unknown)	17/10/2	297/6/14	<0.0001
Treatment approach			
Surgical resection	27	285	0.75
Systemic chemotherapy alone	2	32	
Adjuvant chemotherapy after curative resection			
None	20	197	0.43
5-FU alone	2	7	
5-FU plus oxaliplatin	4	38	

Data are presented as medians (ranges) or numbers

Abbreviations: *CRC* colorectal cancer, *FU* fluorouracil, *MSI-H *high microsatellite instability, *muc* mucinous adenocarcinoma, *por* poorly differentiated adenocarcinoma, *sig* signet ring cell carcinoma, *tub1* well-differentiated tubular adenocarcinoma, *tub2* moderately differentiated tubular adenocarcinoma, *UICC* The Union for International Cancer Control

### Survival outcomes

**Fig. 1:**
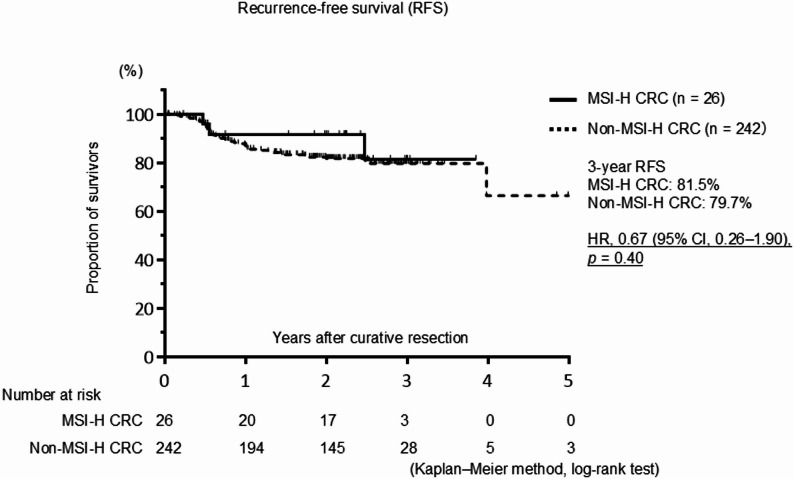
Kaplan–Meier survival curve for recurrence-free survival (RFS) in patients with colorectal cancer (CRC) without distant metastases who underwent curative resection. The RFS curves were not significantly different between patients with high microsatellite instability (MSI-H) and non–MSI-H tumors. Hazard ratio (HR) and confidence interval (CI) values are presented

**Fig. 2:**
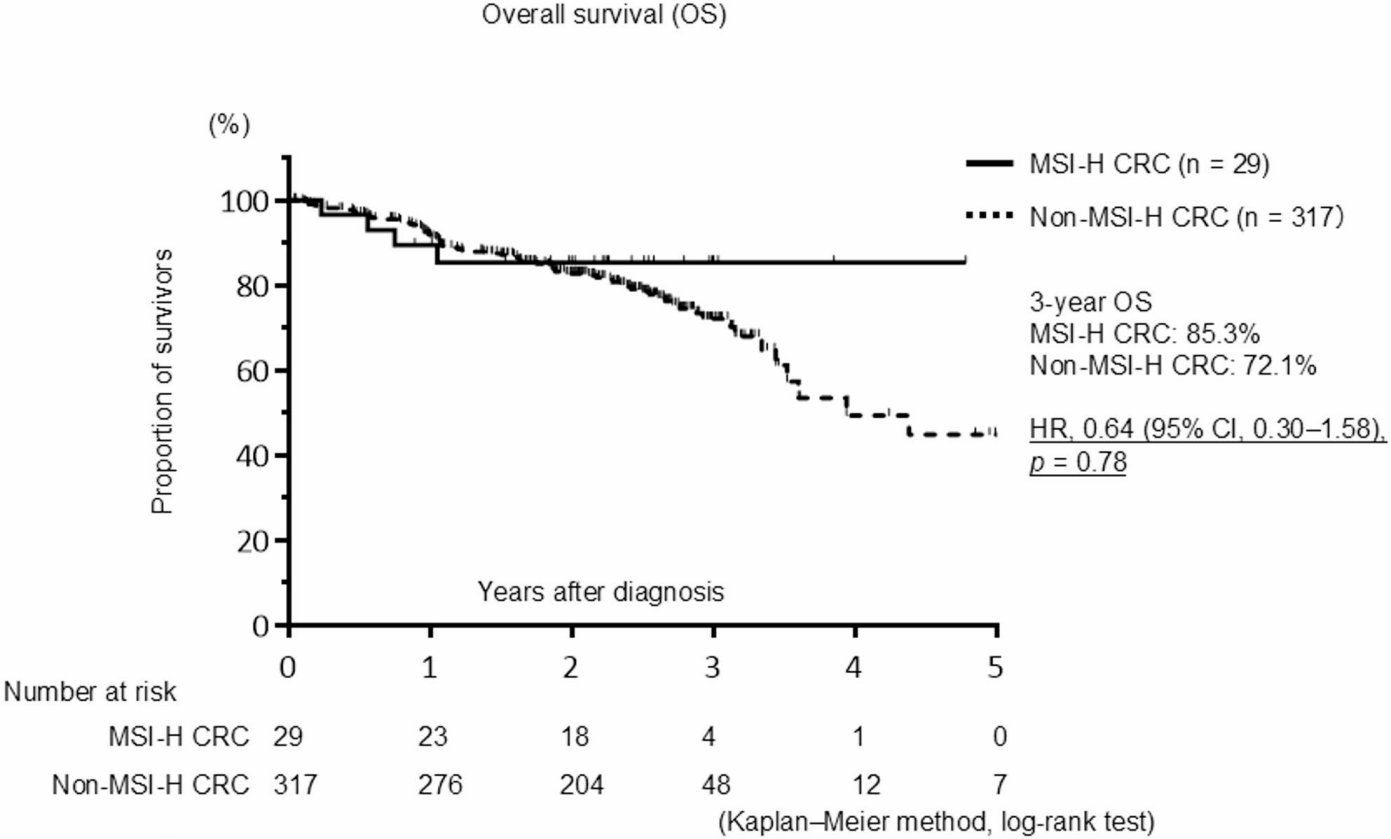
Kaplan–Meier survival curve for overall survival (OS) in all patients with colorectal cancer (CRC), comparing those with high microsatellite instability (MSI-H) tumors to those with non-MSI tumors. The OS curves showed no significant difference between patients with MSI-H and non–MSI-H tumors. Hazard ratio (HR) and confidence interval (CI) values are shown

The median follow-up period was 26.7 months. RFS was analyzed in patients without distant metastasis who underwent curative resection (Fig. 1). Compared with patients with non-MSI-H tumors, patients with MSI-H tumors showed a non-significant trend toward a better RFS (HR, 0.67; 95% CI, 0.26–1.90; *p* = 0.40). Similarly, no significant difference in OS was observed between patients with MSI-H and non–MSI-H tumors (HR, 0.64; 95% CI, 0.30–1.58; *p* = 0.78) (Fig. 2).

### Clinicopathological factors associated with survival

To assess the impact of MSI status on RFS and OS, univariate and multivariate Cox regression analyses were performed, with adjustments for clinically relevant prognostic factors. Multivariate Cox regression analyses identified the following independent prognostic factors: tumor size > 30 mm was associated with worse RFS (HR, 3.63; 95% CI, 1.35–9.75; *p* = 0.01) (Table 2); MSI-H status was linked to favorable OS (HR, 0.07; 95% CI, 0.005–0.90; *p* = 0.04) (Table 3); and *BRAF* mutation was correlated with worse OS (HR, 7.39; 95% CI, 1.32–41.50; *p* = 0.02) (Table 3).

**Table 2:** Univariate/multivariate analyses of factors associated with RFS (Cox regression analyses)

Clinicopathological factors	Univariate			Multivariate		
	HR	(95% CI)	*p*-value	HR	(95% CI)	*p*-value
Age, years (> 75)	1.25	0.71–2.23	0.43	1.12	0.60–2.09	0.72
Sex (male)	1.39	0.77–2.50	0.28	1.20	0.63–2.28	0.58
Tumor location (right)	0.77	0.40–1.49	0.44	0.84	0.39–1.82	0.66
Tumor size (> 30 mm)	4.72	1.86–11.9	0.001	3.63	1.35–9.75	0.01
Tumor depth (T3–4)	3.07	1.21–7.76	0.02	1.51	0.55–4.19	0.42
Lymph node metastases (N1–2)	1.89	1.11–3.25	0.02	1.29	0.68–2.45	0.44
Tumor histology (tub1/tub2)	0.67	0.27–1.70	0.40	1.09	0.32–3.77	0.88
MSI status (MSI-H)	0.67	0.21–2.15	0.49	0.56	0.08–3.69	0.54
RAS status (mutant)	1.12	0.63–1.99	0.69	1.34	0.69–2.59	0.38
BRAF status (mutant)	2.55	1.01–6.46	0.049	4.43	0.99–19.7	0.05
Adjuvant chemotherapy	1.59	0.84–3.03	0.15	1.20	0.56–2.59	0.64

47 recurrence events among 268 patients were observed during the follow-up period

*Abbreviations*: *95% CI* 95% confidence interval, *HR* hazard ratio, *MSI-H* high microsatellite instability, *RFS* recurrence-free survival, *tub1* well-differentiated tubular adenocarcinoma, *tub2* moderately differentiated tubular adenocarcinoma

*p* <0.05

**Table 3:** Univariate/multivariate analyses of factors associated with OS (Cox regression analyses)

Clinicopathological factors	Univariate			Multivariate		
	HR	(95% CI)	*p*-value	HR	(95% CI)	*p*-value
Age, years (> 75)	1.60	1.02–2.51	0.039	1.73	0.79–3.78	0.17
Sex (male)	1.78	1.11–2.86	0.016	1.84	0.82–4.13	0.14
Tumor location (right)	1.18	0.73–1.89	0.51	1.64	0.70–3.87	0.26
Tumor size (> 30 mm)	2.33	1.25–4.35	0.008	1.49	0.59–3.80	0.40
Tumor depth (T3–4)	2.81	1.29–6.11	0.009	1.29	0.42–3.94	0.66
Lymph node metastases (N1–2)	1.82	1.19–2.79	0.006	1.25	0.58–2.68	0.56
Tumor histology (tub1/tub2)	0.80	0.37–1.74	0.57	0.72	0.20–2.64	0.62
MSI status (MSI-H)	0.64	0.23–1.75	0.38	0.07	0.005–0.90	0.04
RAS status (mutant)	1.13	0.72–1.78	0.59	0.84	0.38–1.88	0.68
BRAF status (mutant)	1.71	0.69–4.26	0.25	7.39	1.32–41.50	0.02
Adjuvant chemotherapy	0.69	0.29–1.67	0.41	0.49	0.16–1.53	0.22

78 death events among 346 patients were observed during the follow-up period

*Abbreviations*: *95% CI* 95% confidence interval, *HR* hazard ratio, *MSI-H* high microsatellite instability, *OS* overall survival, *tub1* well-differentiated tubular adenocarcinoma, *tub2* moderately differentiated tubular adenocarcinoma

*p* < 0.05

## Discussion

This study describes distinct clinicopathological characteristics associated with MSI-H status in CRC. Although multivariable analysis suggested a potential association between MSI-H status and improved OS, unadjusted survival analyses did not demonstrate significant differences. These results support the clinical relevance of universal MSI screening within a Japanese cohort of patients with CRC in a real-world setting.

Our results indicate that MSI-H status is significantly associated with right-sided tumor location and poorly differentiated histology, consistent with previous reports [13–16]. Additionally, we observed that patients with MSI-H tumors tended to have a family history of CRC or other cancers, though this association was not statistically significant.

Our genomic analysis further highlights key molecular differences between MSI-H and non-MSI-H tumors. MSI-H tumors exhibited fewer *RAS* mutations but a higher prevalence of *BRAF* mutations compared with non-MSI-H tumors. Notably, the frequent co-occurrence of *BRAF* mutations in MSI-H CRC tumors has been associated with worse prognostic outcomes [17]. However, despite this negative prognostic marker, MSI-H status remained associated with favorable OS, likely because of the influence of immune-related factors. Importantly, our findings highlight the clinical relevance of *BRAF* mutations in stratifying CRC treatment options. Notably, 10 of the 29 MSI-H tumors (34.5%) harbored concurrent *BRAF* mutations, as summarized in Table 1. This molecular overlap is consistent with the established biology of sporadic MSI-H CRC and underscores the heterogeneity within the MSI-H subgroup. The observation that MSI-H status remained associated with favorable OS despite the relatively high prevalence of *BRAF* mutations should be interpreted cautiously. A plausible explanation for this observation is that the immune-enriched tumor microenvironment characteristic of MSI-H tumors may partially offset the adverse prognostic impact of *BRAF* mutations. Alternatively, this finding may reflect limited statistical power and instability of effect estimates due to the small number of MSI-H and MSI-H/*BRAF*-mutant cases, as well as the low number of survival events. Therefore, rather than suggesting a definitive protective effect of MSI-H status over *BRAF* mutation, our results should be regarded as exploratory and hypothesis-generating. Future research encompassing larger samples and including more patients with MSI-H/*BRAF*-mutant tumors, as well as longer follow-up periods, is required to clarify the combined prognostic and therapeutic implications of these molecular features.

The favorable prognostic trends observed in MSI-H CRC in our cohort are consistent with prior studies, adding valuable real-world data from a Japanese population undergoing universal MSI screening [7, 10, 14]. Previous studies have consistently demonstrated that MSI-H tumors are linked to improved survival and lower recurrence rates, largely because of immune-mediated responses and specific tumor biology [2, 7]. By examining these trends in a routine clinical setting, our study enhances the understanding of the prognostic impact of MSI-H beyond controlled clinical trials, thereby supporting its broader applicability as a prognostic marker across diverse clinical environments. Moreover, our findings support the implementation of universal MSI screening for all patients with CRC in daily clinical practice, particularly for the identification of patients with LS.

However, despite its strengths, our study has some limitations. First, genetic testing for *MMR* gene mutations—essential for a definitive diagnosis of LS—is currently not covered by national health insurance in Japan. Consequently, many cases of LS may have been missed in this study, making it difficult to accurately determine the prevalence of LS in the Japanese population. Therefore, the precise proportion of MSI-H cases attributable to LS could not be determined in this cohort. Nonetheless, this limitation reflects a real-world constraint of clinical practice in Japan, rather than a methodological omission of the present study. Despite this limitation, MSI testing remains clinically valuable for guiding treatment decisions [6–8, 18]. Second, the study’s retrospective, single-center design may have introduced selection bias, potentially affecting the generalizability of our findings. Third, although our multivariate analysis identified MSI-H as a potential independent prognostic factor for OS, the small sample size of MSI-H cases (*n* = 29) and the limited number of survival events reduced statistical power and may have compromised the stability of Cox regression estimates. The wide confidence intervals and relatively extreme hazard ratios observed for MSI-H status and *BRAF* mutation may reflect sparse data bias and potential overfitting. Therefore, the multivariable findings should be interpreted cautiously and regarded as exploratory, requiring validation in larger study cohorts with longer follow-up periods. Fourth, while our follow-up period was sufficient to assess early survival outcomes, it may have been too short to fully evaluate long-term survival and recurrence patterns. Future prospective studies with extended follow-up periods across diverse populations are warranted to validate and expand upon our findings.

## Conclusion

This real-world study describes the clinicopathological and molecular characteristics of MSI-H CRC in a consecutive Japanese cohort undergoing universal MSI testing. Although MSI-H status showed a potential association with favorable OS in multivariate analysis, survival differences were not significant in unadjusted analyses, and the results should be interpreted cautiously. These findings support the clinical relevance of MSI testing in routine practice, while highlighting the need for studies with larger samples and longer follow-up periods to clarify its prognostic implications.

## Data Availability

No datasets were generated or analysed during the current study.

## Abbreviations

CI: Confidence interval
CRC: Colorectal cancer
CT: Computed tomography
HR: Hazard ratio
LS: Lynch syndrome
MMR: Mismatch repair
MRI: Magnetic resonance imaging
MSI-H: High microsatellite instability
OS: Overall survival
RFS: Recurrence-free survival
